# An epidemiological study of rates of illness in passengers and crew at a busy Caribbean cruise port

**DOI:** 10.1186/s12889-016-2991-3

**Published:** 2016-04-12

**Authors:** Cathy Ann Marshall, Euclid Morris, Nigel Unwin

**Affiliations:** University of the West Indies, Cave Hill Campus, St Michael, Barbados; Faculty of Medical Sciences, University of the West Indies, Cave Hill Campus, St Michael, Barbados; Epidemiology and Public Health, Chronic Disease Research Centre, University of the West Indies, Cave Hill Campus, St Michael, Barbados

**Keywords:** Cruise ship, Epidemiology, Diseases

## Abstract

**Background:**

The Caribbean has one of the largest cruise ship industries in the world, with close to 20 million visitors per year. The potential for communicable disease outbreaks on vessels and the transmission by ship between countries is high. Barbados has one of the busiest ports in the Caribbean. Our aim was to describe and analyse the epidemiology of illnesses experienced by passengers and crew arriving at the Bridgetown Port, Barbados between 2009 and 2013.

**Methods:**

Data on the illnesses recorded were extracted from the passenger and crew arrival registers and passenger and crew illness logs for all ships and maritime vessels arriving at Barbados’ Ports and passing through its territorial waters between January 2009 and December 2013. Data were entered into an Epi Info database and most of the analysis undertaken using Epi Info Version 7. Rates per 100,000 visits were calculated, and confidence intervals on these were derived using the software Openepi.

**Results:**

There were 1031 cases of illness from over 3 million passenger visits and 1 million crew visits during this period. The overall event rate for communicable illnesses was 15.7 (95 % CI 14.4–17.1) per 100,000 passengers, and for crew was 24.0 (21.6–26.6) per 100, 000 crew. Gastroenteritis was the predominant illness experienced by passengers and crew followed by influenza. The event rate for gastroenteritis among passengers was 13.7 (12.5–15.0) per 100,000 and 14.4 (12.6, 16.5) for crew. The event rate for non-communicable illnesses was 3.4 per 100,000 passengers with myocardial infarction being the main diagnosis. The event rate for non-communicable illnesses among crew was 2.1 per 100,000, the leading cause being injuries.

**Conclusions:**

The predominant illnesses reported were gastroenteritis and influenza similar to previous published reports from around the world. This study is the first of its type in the Caribbean and the data provide a baseline for future surveillance and for comparison with other countries and regions.

## Background

Cruising or travelling by boat or ship for leisure has become a major part of the world-wide tourism industry [1]. The Caribbean cruise industry is one of the largest in the world, responsible for over U.S. $2 billion in direct revenue to the Caribbean islands in 2012 [2]. Over 45,000 people from the Caribbean are directly employed in the cruise industry and 17,457,600 cruise passengers visited the islands in the 2011–2012 cruise year [2].

The main port of entry by sea in Barbados is the Bridgetown port and harbour which also houses the cruise terminal facility. Annually, hundreds of vessels and more than half a million passengers enter the Bridgetown Port. For example, in information given to the study team by the Port Authority, in 2013 alone, 374 vessels, 619,485 cruise ship passengers and 262,947 crew members traversed the Bridgetown Port.

Cruise ships in the modern era can be very large vessels which transport thousands of passengers and crew on a single trip. A typical cruise ship carries 2000 passengers and 800 crew, and the largest ships can have capacities in excess of 5000 passengers and 2000 crew [3, 4]. Outbreaks of infectious disease aboard cruise ships are therefore of public health importance, given that ships are closed or semi-closed settings in which infection may easily be spread and may be difficult to control. This is further compounded by the facts that the average cruise lasts longer than 6 days, there are frequent group activities that increase passenger and crew contact and facilitate the spread of infection, and frequent stops are made where passengers can leave the ship and new passengers and crew can board, providing new reservoirs for infection [1].

Within the past five years epidemic prone diseases such as the pandemic (H1N1) in 2009 which originated in Mexico, the Middle East Respiratory Syndrome Corona Virus which was isolated in 2012 in Saudi Arabia, the Chikungunya virus which emerged in the Americas in Saint Maarten in December 2013 and the Ebola Virus in West Africa in March 2014, were introduced into non-endemic areas by travel [5–9]. These public health events further emphasise that international travel can quickly and extensively affect global health [10–13].

Recent media emphasis on communicable disease in the Caribbean such as Chikungunya and Dengue Fever and the expressed global concern over the Ebola virus has heightened public awareness of the possibility of the spread of infectious disease through visitors to a country at its ports of entry.

Barbados has a public health monitoring system in place for entry at each of its ports which records data on all arriving passengers and crew and maintains a log of all diagnosed illnesses. This study provides an epidemiological description of the illnesses presenting at the Bridgetown Port and cruise terminal from 2009 to 2013, and illness rates of passengers and crew, by time of year, vessel and last port of call. This study is the first published analysis of the illnesses in passengers and crew aboard ships in the Caribbean from the perspective of a port health authority.

## Methods

### Design

This is a retrospective descriptive study of rates of illness, making use of routine data sources of communicable and non-communicable illnesses reported by passengers and crew on vessels within the jurisdiction of the Barbados Port Health Department between January 2009 and December 2013.

Ethical approval to undertake this study was applied for before commencing the data collection in June 2014. Ethical approval was formally obtained by written correspondence from the Institutional Review Board (IRB) of the University of the West Indies on the 18^th^ May 2014.

### Setting

Barbados is an independent nation with 286,000 inhabitants. It is a developing economy [14], a member of the United Nations conference of small island developing states [14], and has an estimated per capita gross national income of 14,880 US dollars [15]. Tourism is the major contributor to the economy. Bridgetown is the main shipping port of Barbados, through which 90 % of the goods used in the manufacturing and retail sectors of Barbados pass, and at which all cruise vessels dock.

The Barbados Port Health Department is the government agency responsible for enforcing the International Health Regulations (2005) and regulating public health conditions on vessels transiting through Barbados’ territorial waters.

This includes ships docking at Bridgetown and other Ports in Barbados. Environmental Health Officers (Port Health) are responsible for documenting sick passengers and crew on all vessels, whether visiting a port or simply passing through Barbados’ territorial waters. They do this by boarding all vessels and receiving from the responsible officer on board the total number of sick passengers and crew at the that time. Details that are collected include age, sex, and type of illness for each sick person. This is done as a legal requirement in compliance with International Health Regulations and the data is routinely collected for all ships and assists with disease surveillance efforts at the Bridgetown Port.

### Data collection, abstraction and management

Data collected on sick individuals on board ship by the Port Health Officers were recorded during the time of the study in hard copy illness log books kept at the Bridgetown Port. The data recorded for each sick person in the log book include date, age, sex, type of illness and the name of the vessel. In a separate register the total numbers of passengers and crew on each vessel at the time of inspection were recorded. It should be noted that there is no breakdown by age and sex of the total numbers of passenger and crew.

Data were extracted from passenger and crew registers and illness logs and entered directly into an Epi Info 7 data entry form. Illness episodes were categorised into communicable and non-communicable, and within each of these into common diagnoses (the categorisation being based on a pilot study) or ‘other’. For each sick individual the following was abstracted: age, sex, name of vessel, number of passengers and crew on the vessel, and last Port of Call. Last Ports of Call were categorised into ports of the Organisation of Eastern Caribbean States, ports of other Caribbean islands, United States of America and all other ports of call.

### Analysis

Epi Info 7 was used for all analyses, with the only exception being the use of Openepi to calculate 95 % confidence intervals on the rates. Crude rates were calculated separately for passengers and crew, and expressed per 100,000 per year. Note that the rate is per 100,000 *visits,* as one individual, such as a crew member, may visit Barbados more than once over the course of a year. In describing the results, we highlight differences between two rates where the 95 % confidence do not overlap. Neither age and sex specific nor age adjusted rates could be calculated as the total numbers of passengers and crew, the denominators for the rates, were not available by sex or age group. Finally, vessels were ranked according to the number of communicable disease cases in passengers over the study period.

## Results

There were a total of 4,859,682 passenger and crew visits to Bridgetown Port and Cruise Terminal or through Barbadian territorial waters during the study period (2009–2013). Passengers and crew arrived on a variety of vessels including, cruise ships, yachts, cargo vessels, tankers, fishing vessels, tugs and research vessels. Seventy percent (70 %) of the visits were by passengers, representing 3,424,324 visits.

An overview of the total passengers and crew for the study period (2009–2013) is shown in Table 1, including the last Port of Call for sick passengers. Over 50 % of sick passengers were aged over 60, with a median age over the study period of 64 years, while the sick crew were much younger with a median age of 31 years (Table 2). The majority (73 %) of the sick crew were male, compared to 48 % in passengers.

**Table 1:** Barbados Port Health Activity, 2009 to 2013

		2009	2010	2011	2012	2013	2009–13
Total passenger visits							
	Number	772593	742137	682123	607986	619485	3424324
	% in low season^a^	30.6	31.1	28.5	27.5	32.3	30.0
Total crew visits							
	Number	341346	301541	276706	252818	262947	1435358
Individual vessels with at least 1 case of illness		42	44	35	28	26	96
Number of cases illness by last Port of Call							
	OECS^b^	184	267	86	100	46	683
	Other Caribbean	13	73	65	53	23	227
	USA^c^	10	5	12	10	2	39
	Other	19	35	9	9	8	80
	Not recorded	0	1	0	0	1	2
Total		226	381	172	172	80	1031

^a^1^st^ May to November 30^th^, ^b^Organisation of Eastern Caribbean States, ^c^Includes Puerto Rico

**Table 2:** Number, median age (interquartile range, IQR), and percentage male of sick passengers and crew (note that total cases are 1030, as one case was not recorded as passenger or crew)

	2009	2010	2011	2012	2013	2009–13
Passengers						
Number	114	269	98	123	51	655
Median age	61	65	62.5	64	65	64
IQR	(36–73)	(47–73.5)	(47–70)	(48–71)	(58–77.5)	(47–72.5)
% male	56.1	42.4	43.9	52.9	51.0	47.6
Crew						
Number	112	112	74	49	28	375
Median age	29	29	29	29	29	31
IQR	(25–38)	(25.5–38)	(25–33.5)	(25–32)	(24–35)	(25.5–35)
% male	76.8	70.5	66.2	79.6	71.4	72.8

The overall event rate for communicable illnesses was 15.7 (95 % CIs 14.4–17.1) cases per 100,000 passengers whilst the overall event rate for crew was higher at 23.9 (21.6–26.7) cases per 100,000 (Tables 3 and 4). Gastroenteritis was the predominant illness experienced by passengers and crew, followed by influenza, and together these accounted for 87 % of all communicable disease events in crew and 93 % in passengers. . The rates of influenza recorded were higher (with non-overlapping 95 % confidence intervals) in the crew than the passengers and the rates of gastroenteritis were similar (with overlapping confidence intervals) in both groups.

**Table 3:** Crude rates per 100,000 passenger visits for communicable and non-communicable illnesses

	2009	2010	2011	2012	2013	2009–13
Communicable						
Gastro-intestinal	7.25	28.84	10.26	16.45	4.68	13.70
Influenza	2.59	0.81	0.44	0.82	0.16	1.02
Other	1.04	2.43	0.59	0.33	0.32	0.99
All	10.87	32.07	11.29	17.60	5.17	15.71
95 % CIs	(8.78–13.46)	(28.25–36.41)	(9.03–14.11)	(14.57–21.26)	(3.66–7.29)	(14.44–17.10)
Non-communicable						
Stroke or MI	1.29	0.94	0.88	0.99	1.61	1.14
Diabetes	0.13	0.00	0.15	0.00	0.00	0.06
Injury	0.00	0.40	0.00	0.33	0.32	0.20
Other	2.33	2.83	2.05	1.32	1.29	2.01
All	3.75	4.18	3.08	2.63	3.23	3.42
95 % CIs	(2.61–5.39)	(2.94–5.93)	(2.01–4.71)	(1.62–4.28)	(2.09–4.99)	(2.85–4.09)

**Table 4:** Crude rates per 100,000 Crew visits for communicable and non-communicable illnesses

	2009	2010	2011	2012	2013	2009–13
Communicable						
Gastro-intestinal	13.77	23.21	18.79	11.87	3.04	14.42
Influenza	12.30	7.96	3.25	3.16	3.42	6.41
Other	5.27	2.65	1.45	3.16	2.66	3.14
All	31.35	33.83	23.49	18.19	9.13	23.97
95 % CIs	(25.94–37.87)	(27.87–41.06)	(18.43–29.94)	(13.64–24.72)	(6.13–13.58)	(21.56–26.64)
Non-communicable						
Stroke or MI	0	0.33	0	0.40	0	0.14
Diabetes	0	0	0	0	0	0
Injury	0	0.66	1.45	0.40	0.38	0.56
Other	1.46	2.32	1.45	0.40	1.14	1.39
All	1.46	3.32	2.89	1.19	1.52	2.09
95 % CIs	(0.63–3.43)	(1.80–6.11)	(1.47–5.71)	(0.40–3.49)	(0.59–3.91)	(1.46–3.03)

It is notable that 61.3 % of all communicable diseases in passengers and crew were accounted for by only ten of the vessels over the five year period (Table 5), with one vessel contributing 27.5 % of all cases.

**Table 5:** Percentages of communicable disease cases in passengers by the ten vessels contributing the largest number. Together these vessels account for 61.2 % of all cases over the 5 year period

Vessel	No. Cases	% of total^b^	Attack rate^a^ per 100,000 (95 % CIs)	
1	147	27.53 %	62.69	(53.34–73.67)
2	33	6.18 %	108.40	(77.2–152.2)
3	31	5.81 %	149.10	(105.1–211.6)
4	23	4.31 %	69.73	(46.5–104.6)
5	18	3.37 %	84.78	(53.6–134.0)
6	17	3.18 %	1011.30	(632.4–1614.0)
7	16	3.00 %	52.48	(32.3–85.24)
8	14	2.62 %	145.36	(86.6–243.9)
9	14	2.62 %	99.60	(59.3–167.1)
10	14	2.62 %	94.45	(56.3–158.5)

^a^Attack rate is based only on the times that the vessel had at least one case of communicable disease, does not include in the denominator passengers on the vessel when there were no cases

^b^Percentage of all communicable disease cases, 2009 to 2013

The overall event rate for non-communicable illnesses was 3.4 (2.9–4.1) per 100,000 passengers with myocardial infarction being the main non communicable illness experienced by passengers. Twenty one passengers died during the five year period, all as a result of non–communicable illness. The overall event rate for non-communicable illnesses among crew was 2.1 (1.5–3.0) per 100,000 with injuries accounting for the majority of illness in this category.

## Discussion

This study aimed to describe the rates of illnesses in passengers and crew at a busy Caribbean cruise port. Such data are scarce, and as far as we are aware, we provide the first published description from a Caribbean port, providing a baseline and comparison for further work.

The predominant type of illness experienced by passengers and crew over the five year period was of a communicable nature. Gastrointestinal illness was the leading cause of illnesses in this category followed by what was recorded as ‘influenza’.

Rates of infectious disease in crew were similar to those in passengers but the crew presented with a higher percentage of influenza. This may be as a result of more ideal conditions for the spread of respiratory infections such as the more confined living quarters that crew members generally occupy [16]. Overall rates of infectious disease in crew and passengers were similar and the pattern of infectious disease is similar to previous published reports, with influenza or influenza-like illness being one of the most significant communicable diseases in maritime health on passenger cruise ships as well as cargo ships [17, 18]. Influenza-like illness outbreaks have been previously reported on cruise ships in Australia, Canada and the USA [19–22].

Outbreaks of gastroenteritis are also well documented occurrences on cruise ships [23–25] and the rates of gastroenteritis were similar in passengers and crew in our study. Even though one may expect to find higher rates of gastroenteritis in the passengers, the similar rates may be as a result of both passengers and crew practising prevention measures such as hand-washing before meals, using hand sanitizers placed in dining areas and the early reporting of symptoms. There were non-communicable illnesses recorded during the study period in both passengers and crew such as diabetes and ischaemic heart disease. The rates of the non-communicable diseases in both passengers and crew were much lower than those for communicable diseases. Acute physical injury rather than chronic illness represented the major category of non-communicable medical conditions in the crew and this likely reflects the fact that crew are generally, on average, younger than the passengers on cruise ships. While we can’t confirm age differences in passengers and crew from our data, not having the ages of all of them, the ages of those experiencing illnesses is clearly very different (Table 2).

It is relevant to note that a relatively small number of vessels provided the majority of cases of communicable diseases, with 10 vessels accounting for over 60 % of all report cases. It is likely that this fact represents a combination of vessel size and frequency of visit to the port. Attack rates did differ markedly between those vessels, with some appearing to favour the spread of communicable disease outbreaks more than others. Better monitoring of outbreaks and attack rates by vessel should make it possible for port health authorities to identify those vessels that may need to review their approaches to preventing and containing outbreaks.

Before concluding we note some of the limitations of this study. These include the fact that we were unable to calculate age and sex specific rates of illness in passengers and crew, as only total numbers were available. We were not able therefore to adjust for differences in age and sex composition between passengers and crew or between different vessels. It was observed as shown in Table 2, that on average passengers are older than the crew and taking age into account may have given greater insight into differences in risk between these two groups. Another limitation is that the data rely on what is reported by the vessels medical officer, and although reporting of cases is a legal requirement it is possible that reporting thresholds differed between vessels and for passengers and crew and under-reporting is therefore a possibility. Finally, we note that when it comes to the comparison of individual vessels, we do not have a record of their number of passengers and crew on the occasions when no cases of illness were reported. We were unable therefore to compare true event rates between individual vessels.

Despite the above shortcomings we believe that we have provided the first description of rates of illness from the perspective of a Port Health Authority in the Caribbean, rather than previous reports which are based on individual vessels. The study therefore provides a baseline for future work from this perspective.

## Conclusion

The findings of this study show a high predominance of infectious diseases such as gastroenteritis and influenza-like illness in both passengers and crew passing through a busy Caribbean cruise port and highlight the need for continuous surveillance at points of entry.

This study is the first of its kind to be undertaken in the Caribbean and provides a base-line for future studies. This is important due to the fact that the Caribbean is a major cruise destination in global terms and the industry is projected to become larger in future years.

Further research is required to determine the origins of the illnesses, whether the illnesses were introduced by boarding passengers, or were endemic among the crew or related to the environment on the vessels.

Having a robust system of disease surveillance, along with periodic and scheduled review of data collected, can play a crucial role in limiting disease spread through the cruise ship terminal. The benefits of sound public health policies in this regard would also potentially benefit the many passengers and crew aboard cruise ships.

### Ethics, consent and permissions

Written ethical approval was obtained from the Institutional review Board of the University of the West Indies on the 18^th^ May 2014. Written consent and approval to extract and use the data for the study was given by the Barbados Port Health Authority and the Ministry of Health in Barbados.

## Abbreviations

IRB: Institutional Review Board
OECS: Organisation of Eastern Caribbean States
USA: United States of America
